# Transcription‐Replication Conflicts: Unlocking New Frontiers in Cancer

**DOI:** 10.1002/bies.70025

**Published:** 2025-06-09

**Authors:** Aleix Bayona‐Feliu, Andrés Aguilera

**Affiliations:** ^1^ Department of Genetics, Microbiology and Statistics, Faculty of Biology Universitat de Barcelona Barcelona Spain; ^2^ Centro Andaluz de Biología Molecular y Medicina Regenerativa CABIMER Universidad de Sevilla Seville Spain; ^3^ Departamento de Genética, Facultad de Biología Universidad de Sevilla Seville Spain

**Keywords:** cancer, chromatin remodelers, DNA damage, genome instability, histone modifications, R‐loops, transcription‐replication conflicts

## Abstract

Genome instability (GIN) is a cell pathology linked to cancer promotion and tumor evolution. Transcription is an essential cellular process but also a potential source of DNA damage and GIN. Transcription‐replication conflicts (TRCs) are a predominant source of GIN, and defective TRC resolution may seriously compromise genome integrity. Importantly, chromatin dynamics helps orchestrate the response to TRCs to preserve genome integrity. Multiple epigenetic deficiencies have been shown to cause transcription‐induced replication stress, resulting in DNA breaks and mutations. Consistently, chromatin alterations are frequent in cancer and correlate with increased mutation burden at TRC sites in tumors. Here, we review our current knowledge of TRC processing, the consequences of its dysfunction, and its relevance in cancer. We focus on the interplay between the DNA damage response (DDR) and chromatin dynamics and discuss the clinical potential of targeting TRCs as anticancer strategies and drugging the associated epigenetic signatures.

## Graphical abstract

Transcription‐replication conflicts (TRCs) constitute a major source of genome instability. Such conflicts are regulated by chromatin, its dysfunction causing replication stress, DNA breaks, and mutations frequently found in cancer. We review the molecular basis of TRCs and chromatin regulation and discuss the clinical potential of targeting TRCs as anticancer therapies.

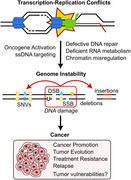

## 1 Introduction

Cancer is a major human health problem that affects millions of people every year. Despite significant advances in therapy research, the disease is still prevalent in our society, and current treatments are often only partially efficient or insufficient to cure the disease. This is in great part due to genome instability (GIN), a hallmark of cancer cells that correlates with high mutagenicity or chromosome rearrangements. These scenarios favor oncogenesis by promoting cancer‐driver gene alterations and adaptation to therapy by providing genetic variation to the tumor, thereby facilitating treatment escape.

Despite being an essential cellular function, transcription may also be a source of DNA damage. While most sources of DNA lesions might only target the DNA occasionally upon exposure to the damaging agent, whether exogenous or endogenous, transcription is active in all cells and thus may become a threat to genome integrity. Current evidence indicates that transcription‐associated genotoxicity arises mostly from transcription‐replication conflicts (TRCs) [1, 2]. As DNA replication and transcription occur on the same template, they have the potential of interfering with each other. These events might be further challenged by the occurrence of transcription‐associated structures that can hinder DNA replication, like non‐B DNA structures such as the R‐loops. These are three‐stranded nucleic acid structures composed of a DNA‐RNA hybrid plus a displaced single strand DNA (ssDNA). Although they form in a regulated manner and exert physiological functions, in many cases they are unscheduled and have the potential of interfering with DNA replication and repair, thus constituting an important source of GIN.

**FIGURE 1:**
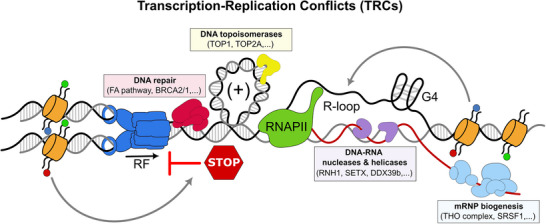
Transcription as a source of genome instability. Transcription uses the same template as DNA replication, and thus has the potential to stall replication fork progression and compromise genome integrity. This effect is further magnified by the occurrence of transcription‐associated structures like non‐B DNA structures such as R‐loops and G4 quadruplexes (G4) or topological stress. To avoid resulting in DNA lesions, cells possess several mechanisms that help prevent and solve transcription‐replication conflicts (TRCs). These include factors involved in mRNP biogenesis that prevent nascent RNA from annealing back to the template DNA, DNA‐RNA helicases and nucleases that degrade and unwind the DNA‐RNA hybrid of R‐loop structure, and DNA topoisomerases that release topological stress. In addition, the DNA damage response (DDR) is highly active at these sites to help preserve genome integrity. Chromatin dynamics influence these processes and contribute to the prevention and proper processing of TCRs and their consequences.

Current data indicate that the DNA damage response (DDR) and its epigenetic regulation are critical at TRCs [3, 4, 5, 6, 7, 8, 9, 10, 11, 12]. Indeed, deficiencies in DNA repair, S‐phase checkpoint signaling, post‐replicative repair (PRR), and chromatin remodeling result in TRC‐associated DNA damage and GIN, which are common in human malignancies. In addition, TRCs correlate with mutation hotspots in cancer, and defective chromatin remodeling further increases the mutation burden at these sites in tumors [3, 13]. These observations suggest that TRCs are a significant source of mutation in tumors and, most importantly, that chromatin alterations further exacerbate the fragility of these sites (Figure 1). Thus, deciphering the genetic interactions between chromatin regulation and DNA repair that help sustain genome integrity at these sites may help reveal additional tumor vulnerabilities that might be of interest in the clinic. Here, we discuss the current knowledge on TRC regulation, its role in oncogenesis, and the benefits and potential biomedical applications of targeting these genetic interactions in cancer therapy.

## 2 TRCs as a Source of GIN

Eukaryotic expression of protein‐encoding genes involves transcription, RNA processing, export, and translation. Tight regulation of these processes ensures cell viability and drives cellular differentiation and cell fate. Epigenetic regulation helps control gene expression by fine‐tuning transcription rates. However, transcription can also compromise genome integrity, as it shares the same DNA template as replication and repair and can interfere with these processes [1, 14]. These adverse effects might be further intensified by the occurrence of transcription‐mediated topological stress or the accumulation of non‐B DNA structures like G‐quadruplexes or R‐loops during transcription (Figure 1). Indeed, there is evidence that unscheduled R‐loops further hamper DNA replication, resulting in DNA breaks and hyper‐recombination, supporting the view that TRCs constitute a major potential source of DNA damage and GIN [15, 16, 17, 18]. Consequently, cells have acquired multiple mechanisms that help prevent or resolve R‐loops, thus ensuring proper gene expression and genome integrity (Figure 1). Indeed, factors involved in messenger ribonucleoprotein (mRNP) biogenesis, like the THO complex, TDP‐43 or SRSF1 [19, 20, 21, 22], DNA topoisomerase I and II [23, 24], DNA:RNA hybrid resolvases like ribonucleases H1 (RNH1), 2 (RNH2) and DICER [9, 25, 26], DNA:RNA helicases like DDX39b/UAP56, DDX5, DDX47, DDX21 or DHX9 and others [27, 28, 29, 30, 31, 32], and DNA repair factors such as BRCA1, BRCA2, the MRN complex, and those of the Fanconi anemia (FA) pathway [4, 7–9, 33] have been shown to actively prevent R‐loop accumulation and R‐loop‐mediated GIN.

**FIGURE 2:**
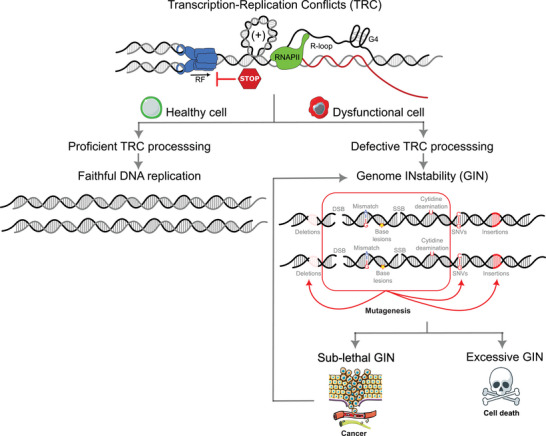
The interplay between transcription‐replication conflicts (TRCs), genome instability (GIN), and tumorigenesis. TRCs are a potential source of DNA damage and GIN. Effective TRC processing ensures faithful DNA duplication and transfer of genetic information to the offspring. However, pathological cells harboring gene alterations challenging TRC homeostasis, like tumor cells, accumulate mutations at these genomic sites. At sub‐lethal levels, TRC‐induced GIN and mutagenesis further contribute to oncogenesis by promoting tumor development and therapy resistance. However, excessive GIN may lead to mitotic catastrophe and cell death.

Current knowledge suggests that GIN helps promote, sustain, and fuel oncogenesis [34, 35]. It is triggered and sustained by replication stress and persistent DNA damage. The fact that DNA‐RNA hybrids and TRCs are a major potential source of DNA breakage provides a potential link between TRC misregulation and cancer proneness (Figure 2). Consistent with this, recent investigations have demonstrated that R‐loop‐associated TRC sites largely correlate with mutation hotspots in cancer cells [3, 13]. Indeed, substantial increases of single nucleotide variants (SNVs), ±1 insertion‐deletion (INDELs), and structural variants (SVs) are present at these sites in tumor samples, particularly at TRC sites occurring in head‐on (HO) orientation, known to be the most detrimental [16, 36–38]. In addition, R‐loop mutagenicity in tumors mostly aligns with defective DNA repair signatures. Deficiencies in homologous recombination (HR), transcription‐coupled nucleotide excision repair (TC‐NER), and DNA mismatch repair (MMR), along with replication slippage, activity of the AID/APOBEC family of cytidine deaminases, and reduced RAD52 expression, are key contributors to the mutagenesis of these sites in cancer [3, 13]. Consistently, recent evidence suggests that the synthetic lethal effect of PARP inhibitors in the context of HR deficiency is primarily mediated through TRCs rather than trapped PARP proteins [39], further supporting a strong association between TRCs, DNA repair deficiencies, GIN, and mutations at these sites in tumors.

These findings suggest that TRCs serve as a source of GIN and mutagenesis in tumors, thereby playing a direct role during oncogenesis and providing a basis for the selective targeting of cancer cells (Figure 2). GIN induced by TRCs might cause genome alterations at oncogenes and tumor suppressors and provide genetic variation to the tumor, potentially facilitating malignant transformation and tumor adaptation, proliferation, and survival. Importantly, however, these events simultaneously create unique tumor‐specific vulnerabilities that can be strategically exploited to selectively target and eliminate malignant cells. This new knowledge opens the possibility of targeting factors promoting or processing TRCs as potential anticancer therapies.

## 3 TRC Regulation and Chromatin Dynamics

TRC prevention and resolution requires a coordinated cellular response that involves mRNP biogenesis factors, DNA topoisomerases, DNA‐RNA helicases and ribonucleases, and DNA repair factors [15, 18, 40]. Increasing evidence indicates that chromatin dynamics exert a key role by helping orchestrate the response to TRCs [15, 41]. Multiple epigenetic factors from a broad range of chromatin regulatory pathways have been shown to help protect forks at TRCs, suggesting the existence of a large regulatory network including the enzymatic activities directly regulating TRCs and the chromatin state (Figure 1).

Indeed, disruption of core components of SWI/SNF ATP‐dependent chromatin remodeling complexes like BRG1/SMARCA4, BAF250A/ARID1A, or ATRX, and key subunits of INO80, ISWI, and CHD remodeling families such as INO80, SNF2H/SMARCA5, or MTA2 induce TRCs and TRC‐associated DNA damage [3, 10–12, 42, 43]. Fine‐tuning of histone turnover and post‐translational modifications (PTMs) are also critical to prevent TRCs and preserve genome integrity. Histone chaperones are crucial to ensure correct transfer and deposition of histones. In this sense, reduced histone H1 dosage and suppression of FACT and HIRA chaperone complexes lead to strong R‐loop‐mediated GIN phenotypes [6, 44–46]. Similar scenarios are observed in cells deficient in chromatin writers, readers, and erasers such as the polycomb repressive complexes (PRCs), the G9a/GLP histone methyltransferase complex, the SIN3A histone deacetylase (HDAC) complex, or the acetylated histone reader BRD4 [47, 48, 49, 50, 51, 52]. DNA and RNA modifications are also emerging as pivotal regulators of R‐loop homeostasis. To date, methylation of CpG islands, methylation of the N6 position of adenosine (m6A) and N5 position of cytosine (m5C) in RNAs, and RNA editing by ADAR RNA adenosine deaminase enzymes have been reported as suppressors of R‐loops, underscoring the critical roles of epigenomics and epitranscriptomics in the turnover of these structures [53, 54, 55, 56, 57, 58]. Consistently, multiple epigenetic factors are enriched at TRCs, particularly at HO TRCs, which are more difficult to resolve. Notably, ATP‐dependent chromatin remodeling and chromatin organization were among the top enriched gene categories at these sites, alongside classical DDR pathways such as replication fork (RF) processing and protection and DNA replication checkpoint signaling [3].

Intriguingly, multiple chromatin activities with a priori opposite functions seem relevant to preserving genome integrity at sites undergoing TRCs. As an example, deficiency of polycomb but also trithorax proteins like SWI/SNF results in TRC‐induced GIN. In the same line, BRD4 is antagonistic to other chromatin activities that contribute to TRC control, such as G9a/GLP, Polycomb, or HDACs. Likely, the epigenetic determinants of genome stability at TRCs might be dependent on the genomic and chromatinic context. Consistent with this, the main ATPases from SWI/SNF, ISWI, and INO80 chromatin remodeling families were found to associate with mutually exclusive and specific R‐loop subsets and top chromatin activities enriched at TRCs, which were found to align with distinct mutational signatures in tumors [3]. In addition, several chromatin enzymes have been shown to functionally interact with distinct and specific regulatory factors. Thus, disruption of SMARCA4, but not SMARCA2, was shown to alter R‐loop metabolism through functional interactions with the FA pathway, despite both being ATPases of the human SWI/SNF family [10]. Similarly, the SIN3A HDAC complex was shown to interact with the THO mRNP biogenesis and export complex to promote chromatin deacetylation and compaction and prevent the nascent RNA from annealing with the template DNA [47].

Unexpectedly, heterochromatin‐associated factors also appear to be highly relevant to preventing TRC‐induced genotoxicity. Despite heterochromatin being generally associated with transcriptional silencing, transcription is crucial for promoting heterochromatinization [59]. Likely, deficiencies in such pathways might lead to cryptic aberrant transcription regulation phenotypes at heterochromatin sites and promote unscheduled R‐loop formation at these sites. For example, histone H1 was shown to specifically induce R‐loop‐dependent DNA damage at heterochromatin, consistent with its main attributed role associated with heterochromatin regulation [45]. Altogether, this indicates that multiple regulatory pathways and chromatin dynamics converge at TRCs, pointing to an extensive interactive regulatory network to safeguard genome safety at these loci.

## 4 TRCs and Cancer

**FIGURE 3:**
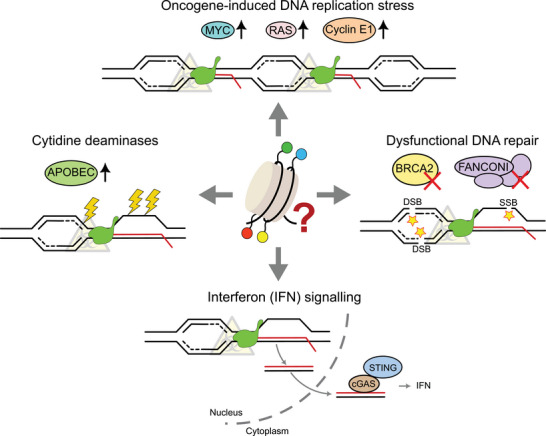
Sources of transcription‐replication conflict (TRC)‐driven GIN in tumor cells. TRCs may arise as a consequence of multiple tumor‐associated features. MYC, RAS, and Cyclin E1 oncogene overexpression has been shown to promote TRCs genome‐wide. Increased expression of cytidine deaminases like APOBEC and AID is common in certain cancers. Higher amounts of these enzymes can promote increased gene editing of ssDNA associated with TRCs. Deficiencies in DNA repair compromise DNA repair during TRCs and favor DNA lesions and mutations at these loci. Finally, TRC‐prone R‐loops have been reported to induce type I interferon (IFN) response through cGAS‐STING pathway. Additional alterations to genes associated with the IFN response could be used by cancer cells to escape the immune response. On top of that, chromatin plays a pivotal role in controlling these processes and could be considered as a potential anticancer target.

Dysregulation of TRCs triggers DNA replication stress and GIN, widely recognized pathological features of cancer cells. Sources of GIN are diverse and can be either endogenous or exogenous, normally requiring direct exposure to toxic compounds or radiation. In contrast, transcription and TRCs constitute a pervasive source of GIN in cells. Notably, alterations in TRC regulatory pathways are common in human malignancies, suggesting the existence of an intricate and multifaceted relationship between TRC‐induced GIN and the processes driving oncogenesis (Figure 3).

First, defects in the DDR are a prevalent characteristic of human malignancies [60]. Alterations in DDR components involved in resolving DNA replication stress are prevalent in tumors, and numerous enzymes involved in DNA repair pathways are cataloged as cancer driver genes according to the Catalogue Of Somatic Mutations In Cancer (COSMIC) [61, 62]. Consistently, a substantial proportion of cancer‐mutated DDR components have been shown to help mitigate TRC toxicity. Indeed, RF progression through R‐loop‐prone genome regions is dependent on BRCA2, BRCA1, and the FA pathway, 9‐1‐1/ATR/CHK1 and MRN complexes [4, 5, 7–9, 33, 63]. Consistent with that, defects in DDR factors participating in RF restart, reversal, and repriming such as RAD52, MUS81, or PRIMPOL, and enzymes involved in RNAP removal, like RECQL5, also challenge RF progression through actively transcribed sites [64, 65, 66, 67]. In addition, abnormal ATM/CHK2 signaling and PRR result in unrepaired DSBs and post‐replicative ssDNA gaps that promote unscheduled DNA‐RNA hybrid accumulations, disrupting DNA replication [5].

Second, oncogene‐induced DNA replication stress is a hallmark of certain tumor types. Overexpression of cyclin E or MYC has been shown to increase the number of RFs by aberrantly activating intragenic origins, thereby enhancing RF collapse at transcriptionally active regions [68, 69]. Likewise, oncogenic RAS increases RNA synthesis genome‐wide, leading to TRC‐induced DNA replication stress and subsequent DNA damage [70, 71]. In line with this, disruption of R‐loop homeostasis by increased TOP1 expression in mutated RAS cells leads to accelerated DNA replication and heightened DNA damage [72].

Third, the displaced DNA strand of an R‐loop structure constitutes an additional hazard, as it can be targeted by multiple enzymes and result in DNA breaks. Despite the protective role of RPA proteins in maintaining genomic stability by coating single‐stranded DNA (ssDNA) and limiting access to its sequence, pathological DNA replication stress can surpass the capacity of these proteins. This results in an insufficient supply of RPA, rendering ssDNA vulnerable and accessible to various nucleases and other proteins [73]. Interestingly, certain nucleases targeting ssDNA are also overexpressed in various cancers, contributing to genomic instability and tumor progression. Thus, flap endonuclease 1 (FEN1) or DNA2 nuclease, which participate in DNA replication and DNA repair, are frequently altered in breast, ovarian, and pancreatic cancers [74, 75]. In line with this, alterations in cytidine deaminases like the apolipoprotein B mRNA‐editing catalytic polypeptide‐like (APOBEC) family of enzymes or activation induced cytidine deaminase (AID) are also frequently observed in specific cancer contexts, and their dysregulation or aberrant activity results in GIN [76, 77]. These enzymes catalyze the deamination of deoxycytidine in ssDNA, converting them into deoxyuridine, respectively. The resulting deoxyuridine is then targeted by uracil‐DNA glycosylases (UDGs), causing ssDNA gaps and further challenging DNA replication and genome safety. Notably, these enzymes have been directly linked to R‐loop metabolism, and the mutational signature induced by these enzymes is highly present at R‐loop‐prone sites in tumors [3, 78].

Finally, pathological R‐loops also induce anti‐tumor immunity. Nucleolytic processing of R‐loops generates ssDNA and DNA‐RNA hybrid sequences that can be sensed by the cGAS‐STING pathway and TLR3 once exported to the cytoplasm and trigger innate immune response [79]. Of note, dysfunctional R‐loop regulation further exacerbates this response, as observed in SETX‐, BRCA1‐, and ARID1A‐mutant cells [79, 80]. It is likely that tumor cells evolve additional mechanisms to evade TRC‐induced immune responses and ensure their survival.

These data suggest that transcription must be tightly regulated through specific compensatory mechanisms to limit its capacity to challenge genome integrity. A good example of that are pediatric cancers, which are known for their transcriptional dependencies while maintaining relatively low mutational burdens [81]. Interestingly, this raises the question of whether they inherently experience fewer transcription‐replication conflicts (TRCs) or if they have evolved mechanisms to better resolve them. One possibility is that pediatric tumors have lower overall TRC levels due to their developmental origins, where chromatin landscapes and transcription programs are tightly regulated, potentially reducing conflicts with replication. Alternatively, they might encounter similar TRC levels as other cancers but compensate more efficiently through robust replication stress response mechanisms or distinct chromatin states that facilitate conflict resolution. Further investigation into this topic could provide valuable insights into the mechanisms that enable cells to manage TRCs despite high transcriptional activity, with potential implications for therapeutic strategies targeting transcription‐associated vulnerabilities.

Altogether, this highlights the multifaceted impacts of TRC‐induced GIN on oncogenesis, including its role in promoting malignancy and enabling immune evasion. However, the potential of targeting transcription‐induced GIN as an anticancer strategy has not been extensively investigated.

## 5 Chromatin Alterations, TRCs, and Cancer

Epigenetic disorders underlie multiple human diseases, including cancer [41, 62, 82]. Mutations in histone‐modifying enzymes such as EZH2, SETD2, KDM2C‐D, KDM6A, and EP300, subunits of ATP‐dependent chromatin remodelers including SMARCA4, ARID1A, SMARCB1, and ATRX, as well as DNA methylation regulators such as DNMT3A or TET2 are well‐established drivers of oncogenesis. Interestingly, multiple epigenetic factors with high mutation rates in cancer play a pivotal role in preventing TRC‐mediated GIN. Moreover, deficient chromatin remodeling has been associated with an elevated mutational burden at sites prone to R‐loop formation in tumors [3]. These evidence are consistent with chromatin alterations fueling GIN via TRCs in cancer and might be related to the increased mutation frequencies observed for certain epigenetic factors in tumors.

Chromatin alterations may exacerbate TRC‐induced GIN and influence tumorigenesis through multiple mechanisms (Figure 3). First, epigenetic alterations promote unscheduled R‐loop formation, enhancing TRCs genome‐wide [41]. In addition to that, dysfunctional chromatin regulation also interferes with the repair of DNA lesions arising during TRCs. For instance, the SWI/SNF chromatin remodeling complex interacts with the FA pathway, BRCA1, TOP2A and RAD52 to limit TRC toxicity and prevent DNA damage derived from such events [10, 11, 83, 84]. Moreover, the DNA replication checkpoint sensor Mec1‐Ddc2 (ATR‐ATRIP) and the chromatin remodeling complex INO80C (INO80 complex) cooperate to dislodge RNAPII during transcription‐mediated DNA replication stress in yeast [42]. Thus, chromatin aberrations promote TRCs via unscheduled R‐loop formation and interfere with the DDR at these loci, triggering DNA damage and gene mutation, thus providing genetic variation to the tumor and facilitating malignant proliferation and treatment resistance phenotypes.

Oncogene‐driven alterations in chromatin state are also well‐documented and play a crucial role in tumorigenesis. MYC overexpression promotes changes in chromatin structure, leading to altered gene expression patterns, and increases cohesin loading on DNA, contributing to replication stress [85, 86]. In addition, oncogenic RAS mutations are associated with DNA methylation changes that alter gene expression and drive cancer, whereas oncogenic overexpression of EGR induces changes in gene expression associated with changes in chromatin [87]. Taken together, these observations suggest that oncogene‐induced aberrant gene expression and origin firing leading to increased TRC‐mediated DNA replication stress in genomic contexts are chromatin‐dependent and require pathological chromatin organization.

In this context, cytidine deaminases are not an exception. The density of mutations induced by APOBEC in cancer increases in early‐replicating, chromatin‐accessible regions, reflecting a strong dependence on DNA accessibility and replication timing [88]. Similarly, AID activity is tightly regulated at the epigenetic level, with mechanisms such as chromatin remodeling and histone modifications working together to limit excessive and off‐target DNA lesions and preserve genomic safety [89]. Chromatin disorders in cancer may facilitate aberrant APOBEC/AID genome targeting via TRCs by favoring the exposure of naked ssDNA to these enzymes.

The type I Interferon (IFN) response, triggered by the cGAS‐STING pathway upon detection of cytosolic DNA and DNA:RNA hybrids, also relies on chromatin remodeling and epigenetic mechanisms [90]. The cellular response to type I IFN response requires dynamic modulation of chromatin states at target gene loci to induce downstream expression of interferon‐stimulated genes (ISGs). Disruption of IFN‐mediated chromatin regulatory mechanisms may compromise immune response and provide a survival strategy for tumor cells undergoing TRC‐mediated GIN. Current evidence indicates that type I IFN response is triggered by pathological R‐loop‐prone scenarios [79, 80]. Under these circumstances, epigenetic alterations interfering with the immune response may be positively selected and help escape the immune system by cancer cells. Collectively, evidence points to an intricate relationship between chromatin dysfunction, GIN, and oncogenesis driven by TRCs that contribute to tumor progression and therapy resistance.

## 6 TRCs as Anticancer Targets

GIN accelerates gene mutation and promotes genetic variation, driving malignant transformation and contributing to genetic and epigenetic heterogeneity within the tumor [34, 91, 92]. The extent of GIN, however, must be limited, as excessively high levels of GIN may result in mitotic catastrophe and death. Multiple current cancer treatments and clinical trials to treat human malignancies have been directly conceived to control GIN via the modulation of its determinants. Indeed, targeting GIN based on specific tumor deficiencies is a widely utilized therapeutic approach for cancer patients today.

Synthetic lethality offers a compelling strategy to selectively target cancer cells harboring specific gene deficiencies. Indeed, drugs designed to disrupt these critical functions are rapidly gaining prominence as a major therapeutic option. Multiple anticancer strategies typically exploit the DDR defects of malignant cells by targeting genes that are synthetic lethal to these deficiencies. PARP inhibitors have gained widespread recognition as an effective synthetic lethality‐based therapy, particularly in the treatment of BRCA2‐mutant cancers and tumors that phenocopy HR deficiency, such as Ewing sarcoma (EWS). Likewise, DDR inhibitors targeting key DNA repair factors such as ATR, ATM, WEE1, and DNA‐PK, as well as compounds that disrupt DNA replication, are commonly used in the clinic to treat HR‐deficient tumors and evaluated in clinical trials [93, 94].

Current evidence suggests that TRC‐driven GIN arises from distinct alterations, primarily involving DNA repair deficiencies, oncogene activation, APOBEC hyperactivity, or the cGAS‐STING pathway induction. These insights highlight the potential for exploiting TRC‐driven GIN through synthetic lethal strategies, underscoring its significant clinical relevance as a therapeutic target. Supporting this concept, a recent study showed that targeting TRCs with the small molecule AOH1996 effectively suppressed tumor growth by promoting the dissociation of PCNA from actively transcribed regions, leading to transcription‐dependent DNA damage [95]. Additionally, TRCs have been identified as key mediators of the synthetic lethality between PARP inhibitors and HR deficiencies [39].

Epigenetic drugs are increasingly prevalent in clinical practice, representing a rapidly evolving therapeutic approach. While numerous chromatin‐targeting agents are already available, the field continues to expand with a new wave of compounds targeting chromatin remodeling complexes [96, 97]. Despite the abundance of druggable chromatin‐related activities, their full therapeutic potential remains largely untapped. Notably, the emerging and intricate relationship between GIN and chromatin regulation in oncogenesis is uncovering a wealth of novel therapeutic targets, which may ultimately help unlock the full potential of these drugs as viable treatment options.

The MLL3/4 H3K4 methyltransferase complex and EZH2, a core component of the polycomb repressive complex 2 (PRC2), recruit the nucleases MRE11 and MUS81 to stalled RFs, promoting fork stabilization and contributing to PARP inhibitor resistance [98, 99]. Conversely, the SIN3A HDAC complex limits MUS81 activity and prevents MRE11‐mediated DNA degradation [100], while BRD4 inhibition induces HR deficiency, effectively re‐sensitizing cells with acquired PARP inhibitor resistance [101]. Moreover, targeting the SWI/SNF complex, a key factor in preventing R‐loop‐mediated GIN, has been shown to enhance tumor sensitivity to PARP inhibitors [102, 103].

Emerging studies are also uncovering patterns of mutual exclusivity between alterations in chromatin regulators and oncogenic drivers, potentially linked to TRC‐driven GIN. In neuroblastoma, MYCN amplification and ATRX mutations, both previously linked to TRC regulatory alterations, rarely co‐occur due to their shared role in driving excessive DNA replication stress [104]. Similarly, the EWS‐FLI1 fusion oncogene, which perturbs R‐loop metabolism, depends on aberrant chromatin remodeling through altered p300 recruitment, a regulator of R‐loop homeostasis [105]. Additionally, KRAS and EGFR mutations are frequently mutually exclusive with DOT1L loss, the sole methyltransferase responsible for H3K79me, a histone mark enriched at TRC‐prone sites [106]. Furthermore, APOBEC mutagenesis is significantly influenced by chromatin context, while cGAS‐STING pathway activation is intricately linked to chromatin reorganization [88, 90].

Intra‐tumoral diversity fosters the emergence of subclonal variants that can evade treatment, augmenting the risk of relapse. Increased mutation rates may promote additional genomic alterations in tumors, which may be positively selected during treatment, allowing a subset of tumor cells to escape therapies, including chemotherapy or immunotherapy. Targeting the potential sources of GIN in tumors might help restrict the adaptive capacity and survival of cancer cells and enhance their sensitivity to current therapies, ultimately maximizing therapeutic efficiency. Therefore, combined therapies targeting TRC‐mediated GIN alongside immunotherapy or chemotherapy should be strongly considered for clinical trials to enhance the effectiveness of current treatments.

Altogether, this suggests that drugs interfering with DNA replication, DDR inhibitors and epigenetic drugs targeting enzymatic activities directly involved in TRC regulation could provide additional strategies to exploit cancer‐specific vulnerabilities, as supported (see Table 1). Furthermore, the widespread clinical use and ongoing trials of these drugs present a unique opportunity to accelerate the development and clinical approval of novel cancer‐selective therapies. Nevertheless, targeting TRC‐induced genotoxicity also presents significant challenges. One concern is the potential unintended consequences of such interventions. Inhibiting epigenetic regulators may unintentionally trigger TRC‐dependent DNA replication stress in healthy cells, potentially leading to mutations that increase cancer risk. Additionally, emerging studies have raised questions about the efficacy and safety of using epigenetic drugs in cancer treatment in the long run. As an example, despite tumors driven by carcinogenic insults being delayed in G9a‐depleted skin cells, they show increased GIN and aggressiveness [107]. Therefore, such therapeutic approaches must be evaluated with caution, aiming to identify a therapeutic window that enables selective tumor eradication while minimizing off‐target undesired effects.

**TABLE 1:** A sample of drugs targeting transcription‐replication conflicts (TRCs) with high therapeutic potential.

Drug category	Drug target	Mechanism of action	Representative inhibitors	Status
DNA Damage Response	PARP	DNA replication‐induced DNA damage.	Olaparib	FDA‐approved
			Niraparib	FDA‐approved
			Talazoparib	FDA‐approved
	ATR	Impaired DNA replication stress response.	Berzosertib	Clinical phase II
			Ceralasertib	Clinical phase III
	ATM	Impaired double‐strand break repair.	AZD0156	Clinical phase I
			Lartesertib	Clinical phase II
	CHK1	Disruption of the S/G2 DNA damage checkpoint.	UCN‐01	Clinically phase I/II
			MK‐8776	Clinically phase II
			CBP501	Clinically phase I/II
	CHK2	Disruption of the G1 DNA damage checkpoint.	Isobavachalcone	Pre‐clinical
			CCT241533	Pre‐clinical
			PV1019	Pre‐clinical
	CDK4/6	G1/S cell cycle arrest.	Palbociclib	FDA‐approved
			Ribociclib	FDA‐approved
			Abemaciclib	FDA‐approved
	PCNA	Disrupted TRC resolution.	AOH1996	Clinically phase I
	SAMHD1	Deregulated dNTP pools.	Lomofungin	Preclinical
			L‐Thyroxine	Preclinical
	Fanconi Anemia	DNA replication fork stalling.	PIP‐199	Preclinical
			CU‐2	Preclinical
DNA homeostasis	TOP I	Topological stress.	Camptothecin	Clinically phase I/II
			Topotecan	FDA‐approved
	TOP II	Topological stress.	Etoposide	FDA‐approved
			Doxorubicin	FDA‐approved
	G4	Stabilization of G4.	Pyridostatin	Preclinical
			BRACO‐19	Preclinical
	dNTP pool	Incorporation of nucleotide analogues into DNA.	Gemcitabine	FDA‐approved
			Fludarabine	FDA‐approved
	DNA crosslinks	DNA intra/inter‐strands crosslinks.	Cisplatin	FDA‐approved
			Mitomycin C	FDA‐approved
Chromatin organization	SMARCA4	Aberrant chromatin remodeling.	PFI‐3	Preclinical
			Camibirstat	Clinically phase I
	G9a	Altered histone methylation.	A‐366	Preclinical
			UNC0642	Preclinical
	HDACs	Altered histone acetylation.	Romidepsin	FDA‐approved
			Vorinostat	FDA‐approved
	BRD4	Disruption of histone acetylation binding.	TEN‐010 (JQ2)	Clinical phase I
			I‐BET762	Withdrawn
			Pelabresib	Clinical phase I/II/III
	ADAR1	Abnormal RNA editing.	Rebecsinib	Preclinical

*Note*: Drugs are grouped according to their targeted cellular process. Mechanisms of action, representative compounds, and clinical status are indicated.

Future research should prioritize mapping the comprehensive atlas of epigenetic determinants that govern genome stability during DNA replication stress, with particular emphasis on TRCs as a potential source of GIN. A deeper understanding of these processes could reveal novel tumor vulnerabilities, thereby enhancing current therapeutic strategies.

## Conflicts of Interest

The authors declare no conflicts of interest.

## Data Availability

Data sharing is not applicable to this article as no datasets were generated or analyzed during the current study.
